# Peer-led live research demonstrations: challenging medical student misconceptions about research

**DOI:** 10.1007/s40037-015-0239-z

**Published:** 2016-01-18

**Authors:** Stuart O’Connor, Alexander Kenneth Clarke

**Affiliations:** Warwick Medical School, University of Warwick, CV4 7AL Coventry, UK

**Keywords:** Live research demonstration, Peer-assisted learning, Undergraduate research

## Abstract

Modern health care provision is now fundamentally evidence based, meaning competency in academic medicine is integral to medical training. The Integrated Academic Training pathway provides focussed training in this area at a postgraduate level but no such provision exists at an undergraduate level. A number of peer-led academic societies have emerged across the UK to provide education and support for undergraduates but there is little evidence about the type of peer-led interventions that are effective. We report here the findings of one such peer-led organization, the Warwick Academic Medicine Society. We found that traditional educational interventions, including didactic lectures and small-group teaching, are effective at inspiring students regarding academic medicine but poor at translating this enthusiasm into sustained involvement in research. We find this disparity to be centred on misconceptions amongst students regarding the time and skills required to meaningfully contribute to a research project. Further, we introduce the concept of the Live Research Demonstration (LRD), a novel peer-led educational intervention which aims to address these misconceptions and improve involvement of students in research. Initial pilots of the LRD concept have shown significant promise and we recommend a larger trial across multiple localities to confirm its educational benefits.

## Introduction

Competency in academic medicine has emerged as a vital component of effective health care delivery, but few clinicians have had formal training in its core principles of critical appraisal and research. Further there is currently a paucity of trainees seeking academic careers, despite early involvement in research being a strong predictor of a future academic career [1]. The introduction of the National Institute for Health Research-funded Integrated Clinical Academic Training (IAT) pathway in the UK has gone some way towards improving the situation at a postgraduate level, but there is currently no provision in this programme for medical students. This has been recognized by the Academy of Medical Sciences, who have been sponsoring interventions to boost student research aspirations through its Wellcome Trust-funded INSPIRE programme since 2013. This programme has led to the development of many student academic medicine societies across the UK and a resultant growth in peer-led interventions for improving undergraduate research engagement. There is, however, very little formal literature about which interventions are effective.

We report here the initial observations developed at one INSPIRE-funded student organization, Warwick Academic Medicine Society (WAcMS), and how this led to the development of a novel educational tool aimed at improving engagement with academic medicine in the coming years.

## Preliminary observations

WAcMS is based at Warwick Medical School, which runs a four-year course with an intake of 177 exclusively postgraduate students. Despite the predominance of prior qualification, there have been relatively poor levels of student research engagement, and this has translated into a low success rate amongst those applying for the Academic Foundation Programme, the first stage of the IAT pathway [2]. It is therefore an ideal environment to assess educational interventions aimed at improving engagement with academic medicine. WAcMS itself was founded in 2012 with the aim of improving academic participation through a series of passive and active educational interventions informed by current thinking [3–5]. Passive interventions included guest lectures, research skills seminars and single-day conferences, whilst our active interventions were journal club workshops and academic networking evenings. After 2 years of activity, we retrospectively analyzed all the feedback from our events. Generally, we found WAcMS events to be both well-attended relative to course size and generating highly statistically significant increases in student desires to engage with research.

Dividing the data by cohort year yielded more surprising results. We found an overwhelmingly larger number of students in the first and second years of their medical degree compared with the third and fourth. This was surprising as it was believed that these events would be more relevant to senior students thinking about their postgraduate career. Further investigation identified a perception amongst senior students that involvement in research was too difficult or time-consuming to be viable alongside their traditional medical studies. This was related to a belief that medical research would involve huge time commitments and required specific technical skills. This misinformation is clearly a pervasive problem as it has been identified in several other recent studies [6, 7].

It also seemed reasonable to assume from the data that this attitude was not present when beginning medical school but developed over time and therefore an appropriate educational intervention could be used to address this at an early stage in medical school.

Unfortunately traditional educational interventions in this area focus on outlining the importance of medical research and teaching students about different types of academic activity (audit, systematic review, ethics etc.). No intervention has been reported that aims to address the misconception that research is not possible within the constraints of a medical degree. We therefore developed a novel peer-led educational intervention to address this previously identified educational need.

## The live research demonstration concept

The intervention we devised was termed a Live Research Demonstration (LRD). It involves carrying out a simple experiment in front of a live audience and by doing so provides a ‘whistle-stop’ tour through the lifecycle of a research project, from idea synthesis to presentation of results. The key to success is the selection of an experiment which is engaging, can be performed quickly in front of a live audience and which outputs results in real-time. To make the demonstration more engaging we incorporated audience participation in as many aspects as possible, including data collection, ethical considerations and statistical analysis. We also incorporated small talks by students already involved in research, during periods of data collection and data analysis, to ensure the demonstration remained engaging. The many shortcomings of such a rapid and underpopulated experiment also provide ample opportunity for students to develop their ability to critically analyze experimental methodology.

## Initial pilots of the LRD intervention

WAcMS has so far piloted two LRDs, both in the context of multi-event conferences. The 2014 Student Wilderness Medicine UK conference provided the setting for our first pilot, and to fit with the weekend’s theme the experiment chosen was a simulation of altitude using an electronic hypoxification machine. Students were provided with simple observational equipment and tasked with tracking the biological effects of high altitude live on A3 graphs posted around the room. As they were doing this, discussions took place regarding ethics, study design and clinical applicability and a student described a recent research expedition they had been involved with. Feedback from the event was extremely positive with many students stating their intention to become involved with such research.

The second LRD was carried out at the 2015 WAcMS annual conference. It was designed to test how good medical students were at judging a patient’s body mass index (BMI) from sight alone, and whether any factors influenced this judgement (such as the student’s age and gender). Ten medical students were randomly selected from the audience and asked to estimate the BMI of six well-known celebrities shown in photographs. The known BMI of the celebrity was then compared with the student’s estimate. Entering these results into a pre-programmed spreadsheet took 10 min, and we used this time to interactively discuss the limitations of the study as well as how to approach the statistical analysis. The pre-programmed spreadsheet automatically produced graphs comparing the BMI differences to the student’s age and gender, whilst at the same time performing the appropriate statistical tests. We then finished with a group discussion of both the results and the clinical ramifications. Of the 18 students who provided adequate feedback, all of them reported that the LRD was a useful educational tool.

## Discussion

The first 2 years of operation of WAcMS have highlighted that student-led research societies can be very beneficial in promoting academic medicine amongst medical students. It has also helped us identify an attitudinal problem amongst medical students regarding research, namely that when they come to convert their enthusiasm into an actual project they reach the conclusion that research is excessively time-consuming and requires excessive technical skills. As a result we have developed a new peer-assisted educational intervention, the Live Research Demonstration (LRD), which aims to remedy these opinions and demonstrate that some research is easily manageable in the context of a student’s wider studies. Preliminary feedback has shown this intervention to be successful and we now strongly believe that this entertaining deconstruction of the academic process can demonstrate to students that they are fully capable of carrying out research at an undergraduate level and in doing so improve student research engagement.

Several important lessons were learned trialling the LRD concept. The first is that the experiment must prioritize speed over complexity and statistical power, as the primary focus is the process rather than the final results. This means that the experiment should be kept as simple as possible. The second is that any numerical collection and statistical analysis needs to be done rapidly to maintain audience attention, and ideally should be pre-programmed. The goal is to stress the simplicity and thus accessibility of the statistical methods used in such a basic experiment, rather than to teach any specific techniques. This leads to the final point, which is that small talks should be delivered whenever full audience participation is not required, for example when a few audience members are charged with recording observations. All these recommendations stem from the fact that the goal of the LRD is not to instruct research skills, but to break down misconceptions in an entertaining fashion.

In conclusion, we believe the Live Research Demonstration to be a powerful tool for improving student research engagement and would recommend a larger trial across other medical schools to assess its true educational benefits.

### Funding

None.

Stuart O’Connor and Alexander Kenneth Clarke are the president and vice-president respectively of Warwick Academic Medicine Society as well as the Warwick Medical School student leads for the Academy of Medical Science’s INSPIRE programme. Stuart O’Connor is also the current National Student Association of Medical Research (NSAMR) Secretary.
